# The Beat

**Published:** 2010-07

**Authors:** Erin E. Dooley

## EWG Issues 2010 Sunscreen Guide

In its fourth report on sunscreen products, the Environmental Working Group recommends only 8% of 500 products tested.^1^ The group reports a surge in products boasting an SPF higher than 50, “which sell a false sense of security” since higher SPF does not necessarily equate to more protection. Several products contained ingredients of potential health concern: retinyl palmitate, which has been linked to accelerated development of skin tumors and lesions, was found in 41% of sunscreens assessed, and oxybenzone, an endocrine-disrupting compound, was found in 60%.

**Figure:**
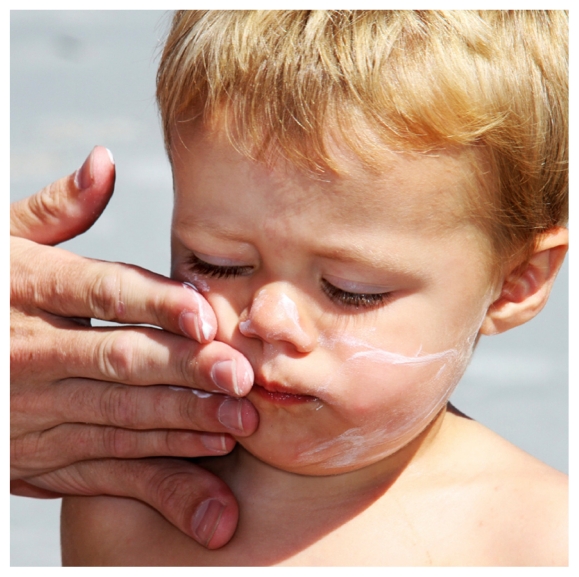
Contrary to public perception, there is no evidence sunscreen prevents skin cancer.

## EPA Exposure Assessment: PBDEs

A new EPA exposure assessment^2^ shows that U.S. exposure to polybrominated diphenyl ethers (PBDEs) occurs primarily through house dust, unlike other persistent organic pollutants, which typically are encountered in food. Additionally, weight-specific intake rates are higher for U.S. children, especially infants, than for adults. The EPA is planning to issue new rules later this year for the manufacture and import of products containing two specific PBDEs. PBDE flame retardants, some of which have already been phased out of commerce, are used in applications including furniture and electronics.

**Figure:**
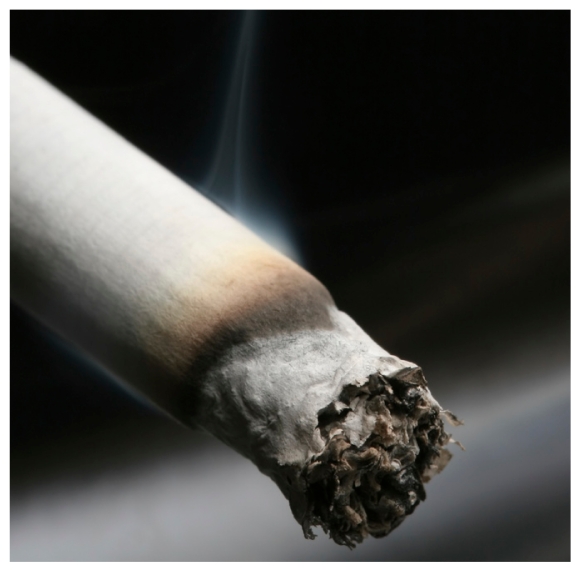
“Safer” cigarettes? Nice try, but no cigar.

## “Safer” Cigarettes Still Hazardous

Smoking tobacco- and nicotine-free cigarettes made of lettuce may be at least as hazardous as smoking conventional tobacco cigarettes, if not more so. In a study of the supposedly safer cigarettes, which were introduced in 1997, dose-dependent double-strand DNA breaks were seen after shorter durations of exposure to smoke compared with conventional cigarettes.^3^ The tobacco- and nicotine-free cigarettes also delivered far higher doses of total particulate matter (“tar”).The researchers used phospho-specific antibodies to measure DNA damage response and their own laser scanning cytometry instrumentation, which they say should be a useful complement to other methods for assessing genotoxicity of cigarette smoke.

## BPA and Male Sexual Dysunction

Bisphenol A (BPA) is used in a large number of consumer products, including plastic containers and food and beverage can linings. Following up on an earlier study^4^ comparing workers with and without occupational BPA exposure, researchers assessed urine BPA levels and sexual function in a subset of workers and found that increasing urine BPA level was associated with decreasing values for seven measures of sexual function.^5^ An additional analysis restricted to workers exposed to BPA only nonoccupationally revealed a similar trend, but the authors wrote that “many of the estimates were no longer statistically significant due to the markedly reduced sample size.”

## Indoor Tanning and Melanoma: Evidence Strengthens

A new study presents strong evidence that use of tanning beds may lead to higher odds of melanoma.^6^ Compared with people who never tanned indoors, people using any tanning bed were almost 75% more likely to develop melanoma, and frequent users of indoor tanning beds had the highest risk. The study also showed for the first time that melanoma was more strongly associated with frequency of tanning than with age at which indoor tanning began. Earlier studies showed only weak associations with melanoma risk; most were unable to adjust for sun exposure or did not confirm dose response or compare specific tanning devices—gaps bridged in the current population-based case–control study. Melanoma, the most dangerous form of skin cancer, is also one of the fastest increasing cancers in the United States.^7^

**Figure:**
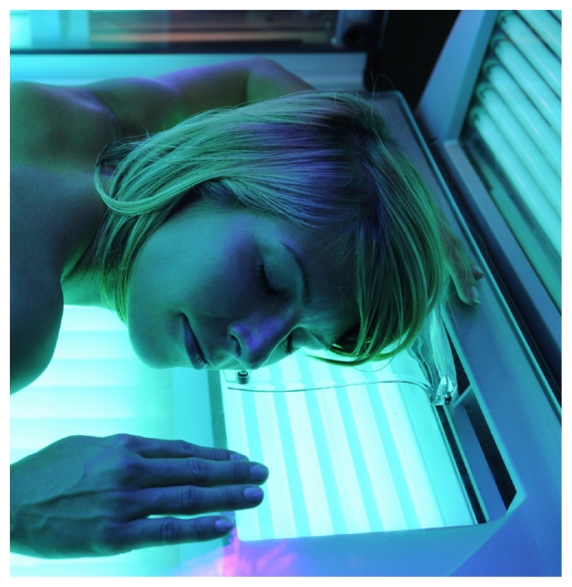
An advisory panel to the FDA has recommended restrictions on the use of tanning beds by teenagers.
